# Differential expression and localisation of TGF-β isoforms and receptors in the murine epididymis

**DOI:** 10.1038/s41598-020-57839-5

**Published:** 2020-01-22

**Authors:** Allison Voisin, Christelle Damon-Soubeyrand, Stéphanie Bravard, Fabrice Saez, Joël R. Drevet, Rachel Guiton

**Affiliations:** GReD laboratory, CNRS UMR 6293 - INSERM U1103 - Université Clermont Auvergne, 28 place Henri Dunant, 63001 Clermont-Ferrand Cedex, France

**Keywords:** Transforming growth factor beta, Infertility

## Abstract

Testes produce spermatozoa that transit through and are stored in the epididymis where they acquire their fertilising capacities. Spermatozoa appear in the genital tract at puberty, long after the immune system was trained to self-antigens. As a consequence, this organ has to set strategies to tolerate sperm antigens to avoid autoimmune responses that would specifically target and destroy them. A recent study pointed the Transforming Growth Factor-beta (TGF-β) signalling in the dendritic cells as a crucial mechanism for epididymal tolerance to spermatozoa. In the mouse, TGF-β exists under three isoforms, and three distinct receptors have been described. Using RT-qPCR, immunohistochemistry and ELISA techniques, we investigated the expression and spatial distribution of the epididymal TGF-β isoforms and of their receptors in young and adult mice. We showed that both ligands and receptors were produced by immune and non-immune cells in the epididymis, whatever the age mice have. These data bring new clues as to the mechanisms of peripheral tolerance to sperm cells in the murine epididymis and raise potential other implications of the cytokine isoforms.

## Introduction

After being produced in the testis, spermatozoa transit through and are stored in the epididymis in order to become fully mature. Therefore, the epididymis has a vital role in managing the constant influx of spermatozoa that appear at puberty. The ability to discriminate self-antigens from foreign antigens relies on the tolerance mechanism which encompasses both central and peripheral tolerances. Central tolerance is established before birth or during the perinatal period in mammals, that is long before the production and release of antigens associated with the various stages of sperm development. In this respect, spermatozoa appear as “foreign” to the adult immune system, and are thus managed by peripheral tolerance which is maintained throughout life. This mechanism relies on interactions between antigen-presenting cells and T cells under tolerising conditions, *i.e*. in the absence of mandatory co-stimulatory signals or in the presence of immunoregulatory cytokines, ligands, leucocyte subsets, or through a combination of these elements^1^. These tolerising interactions result in responses ranging from the destruction of antigen-specific T cells to the generation of actively immunosuppressive antigen-specific T cell subsets^1,2^. Maintaining peripheral tolerance to sperm cells in the epididymis is crucial for the host to avoid the development of autoimmune reactions such as the production of anti-sperm antibodies (ASAs) which are involved in infertility, by preventing natural sperm/oocyte fusion, and even by interfering with *in vitro* fertilisation^3–6^. Among the well-known tolerising molecules, the transforming growth factor-beta (TGF-β) has proven to be a key cytokine^7^.

The TGF-β superfamily comprises more than 30 members, including TGF-β isoforms, bone morphogenetic proteins (BMPs), growth and differentiation factors, activins/inhibins, NODAL and the anti-Müllerian hormone (AMH). They are involved in the regulation of numerous cellular processes, including differentiation, proliferation, cytoskeletal organisation, adhesion, and apoptosis^8^. Three isoforms of TGF-β have been identified in mammals: TGF-β1, TGF-β2 and TGF-β3. Although the isoforms are mostly described as functionally overlapping *in vitro*, isoform-specific knockout mice revealed non-redundant phenotypes. Half of *Tgf-β*1 knockout mice typically die prenatally due to yolk sac defects and the survivors develop inflammatory disorders and eventually die within a few weeks after birth^9–11^. *Tgf-β*2 knockout mice display a wide variety of defects in the development of skeletal, cardiac, lung, eye, spinal, inner ear and urogenital tissues resulting in perinatal mortality^12^. *Tgf-β*3 knockout mice also die perinatally due to developmental defects of the lung and cleft palate^13,14^. TGF-βs are secreted as non-covalent complexes associated with latency-associated peptides (LAPs). After activation by the release of LAPs, TGF-βs trigger intracellular signalling *via* their binding to the TGF-β receptor complex, a tetrameric structure composed of two type I TGF-β receptors (TGF-βRI) and two type II TGF-β receptors (TGF-βRII). A third receptor exists, the TGF-βRIII (also called β-glycan), which is required for an optimal TGF-β2 signalling by enhancing its initial binding to the TGF-βRII. Subsequently, TGF-βRII phosphorylates the cytoplasmic domain of TGF-βRI, triggering the recruitment of the intracellular receptors SMAD2 and SMAD3 to the cytoplasmic domain of activated TGF-βRI, and inducing the phosphorylation of the SMAD2/3 complex. Once phosphorylated, SMAD2/3 forms a trimeric structure with SMAD4, which translocates to the nucleus to activate or repress gene expression *via* binding to SMAD-responsive regulatory regions^15^.

Few studies have explored the presence of TGF-βs in the epididymis. TGF-β1 proteins were shown in the rat epididymis, under a latent form, while active TGF-β3 proteins have been detected in the *corpus* epididymis. *Tgf-β*2 mRNA was not detected, whatever the epididymal region considered^16^. Another study described TGF-β1 and TGF-βRII in the epididymis of the marmoset monkey^17^. While the TGF-β1 was found in the apical cells of the *caput* and *corpus* epididymis, its receptor was found in the principal cells of the same regions, suggesting a paracrine mode of action^17^. Finally, the mouse epididymis presented with high *Tgf-β*1 mRNA levels in the *caput* and *corpus* regions^18^. A recent work has revealed that the loss of TGF-β signalling in dendritic cells (DCs) induced a severe epididymal pathology characterised by leucocytosis with granulomas, generation of ASAs and upregulation of pro-inflammatory pathways, thus indicating a key role for the TGF-β signalling in the establishment of peripheral tolerance to spermatozoa in the murine epididymis^19^.

Considering the lack of knowledge on the TGF-β and receptor isoforms in the murine epididymis, and given the recent revelation of their importance in sperm tolerance, we undertook this work to localise them in the different regions of the organ and to identify the TGF-β-producing cells from pre-pubertal to adult mice. These data give clues concerning the mechanisms set in the murine epididymis to maintain an efficient peripheral tolerance to sperm cells while opening to potential other roles of the cytokine isoforms in the physiology of the organ.

## Results

### Quantification and localisation of TGF-β isoforms in adult mice

**Figure 1:**
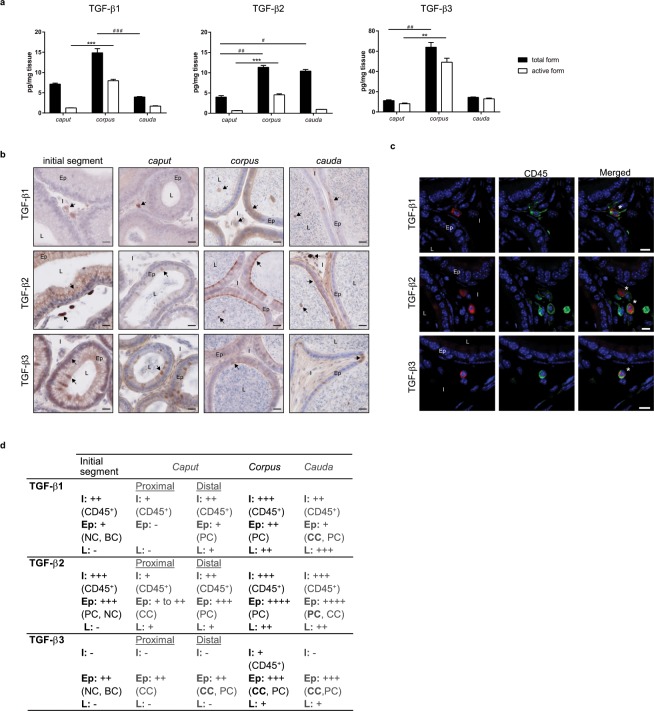
Quantification and localisation of the TGF-β isoforms in adult mice. (**a**) Concentrations of the total and active forms of the three TGF-β isoforms were determined by ELISA in the *caput*, *corpus*, and *cauda* epididymides of 5 month-old mice (pg/mg tissue). Bars represent means ± SEM. n = 7 (TGF-β1 and β2), n = 5 (TGF-β3). Kruskal-Wallis test. ^#^Comparison between total forms: ^#^P < 0.05, ^##^P < 0.01, ^###^P < 0.001. ^*^Comparison between active forms: ^**^P < 0.01, ^***^P < 0.001. (**b**) Localisation of the TGF-β isoforms in the initial segment, *caput*, *corpus*, and *cauda* epididymides of adult mice. Arrows point to representative stained cells. L: lumen, Ep: epithelium, I: interstitium. Scale bars represent 20 µm. n = 10. (**c**) TGF-β-producing cells were identified based on their expression of the surface marker CD45 (pan-leucocyte, green). This immunostaining was done in combination with each isoform of TGF-β (A555, red). Only the *corpus* epididymis is shown and is representative of all segments of adult mice. *Show co-stained cells. L: lumen, Ep: epithelium, I: interstitium. Scale bars represent 10 µm. n = 5. (**d**) Summary of the immunohistochemistry results presented in (**b**,**c**). I: interstitium, Ep: epithelium, L: lumen, BC: basal cells, CC: clear cells, NC: narrow cells, PC: principal cells. −: absence of positive cells, + : low number of positive cells, ++: moderate number of positive cells, +++: high number of positive cells, ++++: very high number of positive cells. Cell density was visually determined.

The concentration of total and active forms of the three TGF-β isoforms was determined by ELISA in *caput*, *corpus* and *cauda* epididymides of adult mice (Fig. 1a). Although all isoforms were expressed in each epididymal region, preferential distributions were observed. Indeed, focusing on the active form of the cytokine, which is biologically relevant, the *corpus* epididymis consistently showed the highest concentrations of TGF-β1, -β2 and -β3. On the contrary, the *caput* epididymis appeared to have the lowest concentrations of the TGF-βs. Moreover, differences exist between isoforms. Interestingly, TGF-β3 was the predominant isoform whatever the epididymal region, followed by TGF-β1 and finally TGF-β2 (Supplementary Fig. S1).

To complete this quantitative analysis, the localisation of each TGF-β isoform was determined by immunohistochemistry. These observations confirmed that the three isoforms were present in every region of the organ even if marked differences were seen between compartments (Fig. 1b,d). Indeed, the interstitial space was populated by TGF-β1 and TGF-β2-positive cells from the initial segment to the *cauda* epididymis while TGF-β3-positive cells were only detected in the *corpus* interstitium (not shown on the selected picture). As the immunosuppressive functions of TGF-β are mostly mediated by immune cells, we tried to identify the interstitial TGF-β-producing cells by immunofluorescent staining using the pan-leucocyte marker CD45 (Fig. 1c,d). For clarity and space reasons, only the *corpus* epididymis staining is shown, as it was representative of all the regions. Finally, all interstitial TGF-β-positive cells, whatever the isoform, were also positive for the CD45 staining, indicating their leucocytic nature.

Epithelial cells also appeared to contain the three isoforms of TGF-β, with the exception of the proximal part of the *caput* epididymis for the TGF-β1. These epithelial TGF-β−producing cells were further identified on the basis of their expression of specific markers and on their tissue location (Fig. 1d and Supplementary Fig. S2). The Supplementary Fig. S2 only shows TGF-β2/AQP9 and TGF-β2/V-ATPase co-staining but the same work was done with TGF-β1 and TGF-β3 (not shown). Results are summarised in Fig. 1d. The principal cells, which present the apical membrane aquaporin 9 (AQP9) on their surface, expressed the three isoforms of TGF-β from the *caput* to the *cauda* epididymis. They also expressed TGF-β2 in the initial segment. The clear cells, which present the proton pump V-ATPase on their apical membrane, are normally found throughout the organ with the exception of the initial segment. Each TGF-β isoform revealed a unique distribution pattern in these cells along the organ: TGF-β1 in the clear cells in the *cauda* epididymis, TGF-β2 in the proximal *caput* and *cauda* regions, and TGF-β3 from the proximal *caput* to *cauda* regions. The narrow cells, which also have membrane V-ATPase, can be discriminated from clear cells by their exclusive location in the initial segment^20^. They expressed the three TGF-β isoforms. Finally, the term basal cells used here refers to cells in basal position in the epithelium. These cells only expressed TGF-β1 and TGF-β3.

The lumen showed no TGF-β-positive cells in the initial segment. It was also the case in the proximal part of the *caput* epididymis, with the exception of TGF-β2-positive cells. Subsequently, the three isoforms of TGF-β were observed in the luminal cells in the *corpus* and *cauda* epididymis (Fig. 1d and Supplementary Fig. S2). Contrary to the interstitial cells, none of the TGF-β-positive intraluminal cells were leucocytes (not shown).

### Expression of TGF-β receptors in adult mice

**Figure 2:**
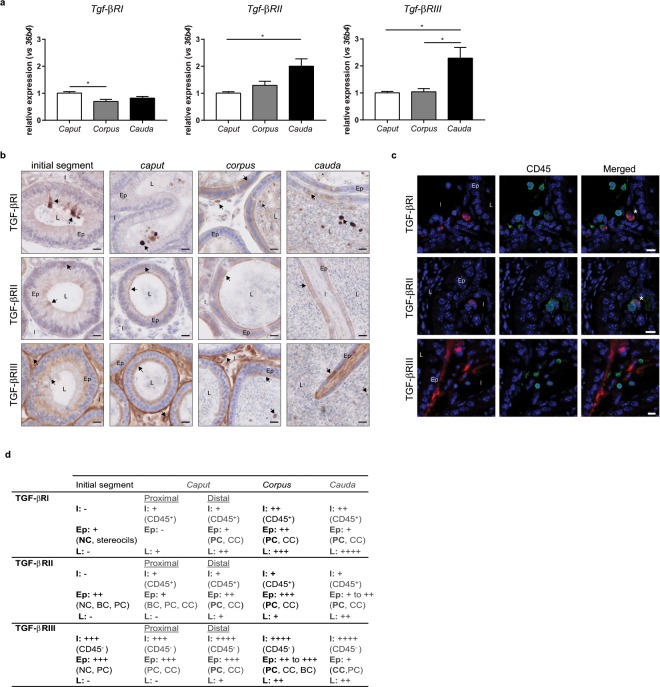
Expression and localisation of the TGF-β receptors in adult mice. (**a**) mRNA expression of the three *Tgf-β* receptors in the *caput*, *corpus*, and *cauda* epididymides of 5 month-old mice. *36b4* was used to normalise the results. Bars represent means ± SEM. n = 6, Kruskal-Wallis test, ^*^P < 0.05. (**b**) Localisation of the TGF-β receptors in the initial segment, *caput*, *corpus*, and *cauda* epididymides of adult mice. Arrows show representative stained cells. L: lumen, Ep: epithelium, I: interstitium. Scale bars represent 20 µm. n = 10. (**c**) Cells positive for TGF-β receptors were identified based on their expression of the surface marker CD45 (pan-leucocyte, green). This immunostaining was done in combination with each isoform of TGF-β receptor (A555, red) in the *corpus* epididymis (representative of all segments of adult mice). *Show co-stained cells. L: lumen, Ep: epithelium, I: interstitium. Scale bars represent 10 µm. n = 5. (**d**) Summary of the immunohistochemistry results presented in (**b**,**c**). I: interstitium, Ep: epithelium, L: lumen, BC: basal cells, CC: clear cells, NC: narrow cells, PC: principal cells. −: absence of positive cells, + : low number of positive cells, ++: moderate number of positive cells, +++: high number of positive cells, ++++: very high number of positive cells. Cell density was visually determined.

To determine if the epididymal TGF-β can trigger local signalling pathways, the mRNAs corresponding to the three TGF-β receptors (TGF-βR) were studied by RT-qPCR in adult organs (Fig. 2a). As the ligands did, the three receptors proved to be expressed throughout the epididymis but with regional discrepancies. *Tgf-βRI* was predominantly expressed in the *caput* epididymis while *Tgf-βRII* and *Tgf-βRIII* were mainly expressed in the *cauda* epididymis.

The receptors were then localised by immunohistochemistry (Fig. 2b,d). As for the ligands, all receptors were found throughout the epididymis, although preferential distributions were observed. The interstitial cells that expressed both TGF-βRI and TGF-βRII in the three regions were immune cells, as showed by CD45 co-staining. On the contrary, the interstitial cells expressing TGF-βRIII were not immune cells (Fig. 2c,d).

Epithelial cells expressed the three receptors with the exception of these from the proximal *caput* epididymis which did not express TGF-βRI. Surprisingly, the epithelial TGF-βRIII staining differed from all the others as it appeared as intracellular granules, whatever the region considered (Fig. 2b). Similarly to what was done for each TGF-β isoform, the epithelial cells positive for receptors were identified thanks to co-staining with AQP9 or V-ATPase (not shown). Results are summarised in Fig. 2d. The principal and clear cells were shown to express the three receptors from the *caput* to the *cauda* epididymis. Basal cells were positive for TGF-βRII in the initial segment and proximal *caput* epididymis and for TGF-βRIII in the *corpus* region. Finally, narrow cells expressed the three receptors in the initial segment.

Luminal cells appeared positive for TGF-βRI in the *caput*, *corpus*, and *cauda* epididymides but not in the initial segment, and their number raised from the *caput* to the *cauda* compartments. Luminal cells positive for TGF-βRII and TGF-βRIII were only observed from the distal *caput* to *cauda* regions (Fig. 2b,d).

Finally, a strong staining for TGF-βRIII was found in the peritubular layer all along the epididymis (Fig. 2b).

### The TGF-β signalling pathway in adult mice

**Figure 3:**
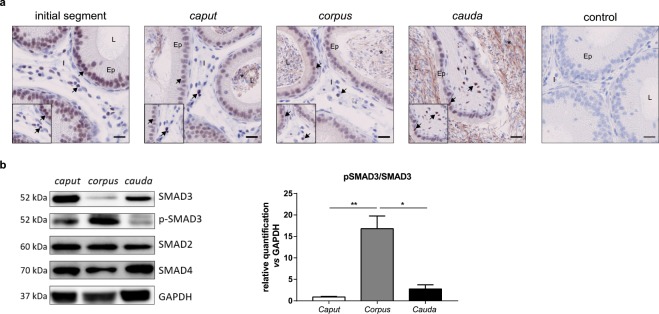
TGF-β signalling pathway in adult mice. (**a**) Immunohistochemistry showing the phosphorylated form of SMAD3 in the epididymis of adult mice. Scale bars represent 20 μm. n = 5. Arrows show representative stained cells. *Show a non-specific staining observed in spermatozoa. L, lumen; Ep, epithelium; I, interstitium. (**b**) Western blot analysis of the TGF-β signalling pathway proteins in the *caput*, *corpus*, and *cauda* epididymides of adult mice (left panel). Blots cropped from different gels are shown. Uncropped versions of the blots are available in Supplementary Fig. S3a. Relative quantification of the ratio between SMAD3 and its phosphorylated form (pSMAD3) normalised with GAPDH (right panel). Bars represent means ± SEM. n = 6, Kruskal-Wallis test, ^*^P < 0.05, ^**^P < 0.01.

As both ligands and receptors were found in the murine epididymis, the activation status of the TGF-β signalling pathway was assessed. The pathway was activated throughout the organ, as illustrated by the presence of the phosphorylated form of SMAD3 (p-SMAD3) in nuclei of both epithelial and interstitial cells (Fig. 3a). A non-specific staining was observed on spermatozoa tails. The TGF-β signalling pathway was then assessed by Western blot analyses that revealed the presence of the downstream actors of the pathway, *i.e*. SMAD2, SMAD4 and p-SMAD3, in the three regions of the epididymis (Fig. 3b, left panel). No phosphorylation of SMAD2 was observed whatever the region considered (not shown). In agreement with the highest concentration of active TGF-β ligands in the *corpus* epididymis, the TGF-β signalling pathway was significantly more activated in this region compared to the other compartments, as illustrated by the quantification of the p-SMAD3/SMAD3 ratio (Fig. 3b, right panel).

### Ontogeny of the TGF-β isoforms

**Figure 4:**
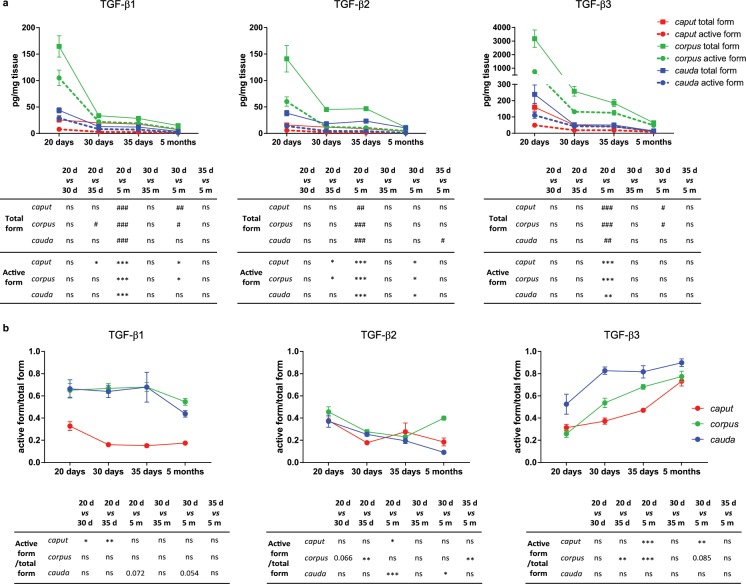
Quantification of the TGF-β isoforms in young mice. (**a**) Concentrations of the total and active forms of the three TGF-β isoforms were determined by ELISA in the *caput*, *corpus*, and *cauda* epididymides of young mice, aged from 20 days to 35 days (pg/mg tissue). To facilitate the understanding, results of adult mice (5 months) from Fig. 1 are also reported. Bars represent means ± SEM. 20 days: n = 6 (TGF-β1 and β2), n = 5 (TGF-β3); 30/35 days: n = 7 (TGF-β1 and β2), n = 5 (TGF-β3); 5 months: n = 7 (TGF-β1 and β2), n = 5 (TGF-β3). Kruskal-Wallis test. ^#^Comparison between total forms: ^#^P < 0.05, ^##^P < 0.01, ^###^P <0.001. ^*^Comparison between active forms: ^*^P <0.05, ^**^P <0.01, ^***^P <0.001. ns: not statistically significant. (**b**) On the basis of data shown in (a), active form/total form ratios were calculated. Kruskal-Wallis test. ^*^P < 0.05, ^**^P <0.01, ^***^P < 0.001. ns: not statistically significant.

In order to provide clues on the role of TGF-β in establishing or maintaining tolerance in the murine epididymis, we quantified the three isoforms in young mice. Based on microscopic observations, we chose to study 20 day-old mice as pre-pubescent animals, 30 day-old mice as mice with newly reached sperm (early puberty, *caput* region only), and 35 day-old mice with sperm that are already passing through the epididymis (pubescent animals). The quantification of the three isoforms showed a similar expression profile over time. Indeed, TGF-β concentrations tend to decrease between 20 days and 30 days and are significantly reduced between 20 days and 5 months. No significant increase in TGF-β concentrations was noted when the sperm cells reach the organ (30 days) or when they pass through it (35 days). Moreover, the *corpus* epididymis was shown to contain the highest concentrations of TGF-β, whatever the isoform, while the *caput* epididymis contained the lowest. Finally, TGF-β3 had the highest concentrations in all segments (Fig. 4a). These two findings were reminiscent of the situation in adult mice (Fig. 1). When considering the ratio of active to total form, it was constant from day 20 to day 35 for TGF-β1 in the *corpus* and *cauda* epididymis (≥0.6) and tended to decrease by 5 months (Fig. 4b). In the *caput* region, a significant decrease was observed between days 20 and 30/35 (0.3 to 0.15). Looking at the TGF-β2 ratios, they were under 0.43 in all regions. A significant decrease was noted in the *caput* and *cauda* epididymis between day 20 and 5 months, while in the *corpus* region, there was a significant decrease from day 20 to day 35 followed by a significant increase at 5 months. It is interesting to note that TGF-β3 ratios increased steadily from day 20 to 5 months in each region. It should be noted that it almost reached 1 in the *corpus* and *cauda* epididymides of adult mice, which means that almost all TGF-β3 present in these regions was in an active form.

**Figure 5:**
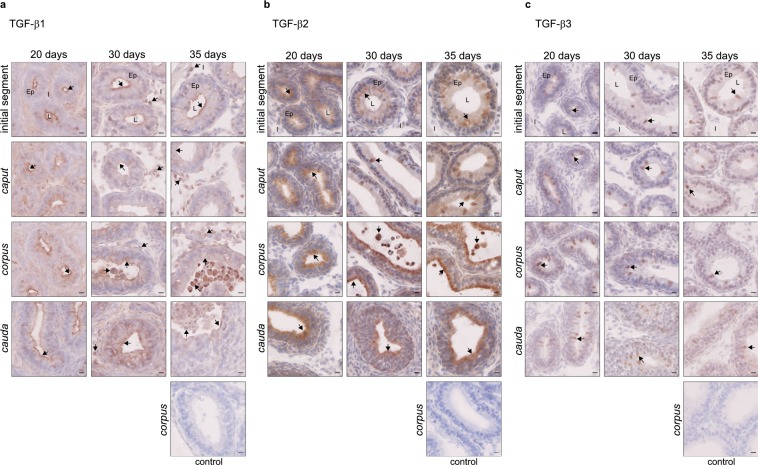
Localisation of the TGF-β isoforms in young mice. Localisation of the TGF-β isoforms in the initial segment, *caput*, *corpus*, and *cauda* epididymides of young mice, aged from 20 days to 35 days. (**a**) Immunohistochemistry showing TGF-β1-positive cells. (**b**) Immunohistochemistry showing TGF-β2-positive cells. (**c**) Immunohistochemistry showing TGF-β3-positive cells. The control pictures refer to the *corpus* epididymis from mice aged 35 days. Arrows point to representative stained cells. L: lumen, Ep: epithelium, I: interstitium. Scale bars represent 10 µm. 20 days and 30 days, n = 5; 35 days, n = 4.

The three isoforms were then localised by immunostaining. All were expressed from the age of 20 days, throughout the organ. TGF-β1 was observed mainly in epithelial cells near the lumen, and some positive luminal cells appeared as early as day 30 (Fig. 5a). An increasing number of positive interstitial cells were observed between day 20 and 35, which were mainly not immune cells (CD45^-^, Supplementary Fig. S4a). This differs from adult mice that had only interstitial immune cells producing TGF-β1 (Fig. 1c). Positive TGF-β2 cells were found in the epithelium from day 20, but staining was changed in the initial segment from day 30 (from homogeneous to granular). Positive luminal cells appeared on day 30 and increased on day 35 (Fig. 5b). No TGF-β2-producing cells were observed in the interstitium of young mice, which strongly differs from adult mice with high levels of TGF-β2-positive interstitial immune cells (Fig. 1c). TGF-β3 was observed mainly in epithelial cells from day 20, although some positive luminal cells were present. While a single cell type appeared to secrete TGF-β3 on day 20 (clear cells), a diversification of the cell types secreting TGF-β3 appeared clearly on day 30 (clear cells, basal cells, narrow cells, not shown). Nevertheless, these cells were different from the TGF-β3-secreting cells in adult mice (Fig. 5c and Supplementary Fig. S4a). Finally, no interstitial TGF-β3-positive cells were observed in young mice.

### Ontogeny of the TFG-β receptors

**Figure 6:**
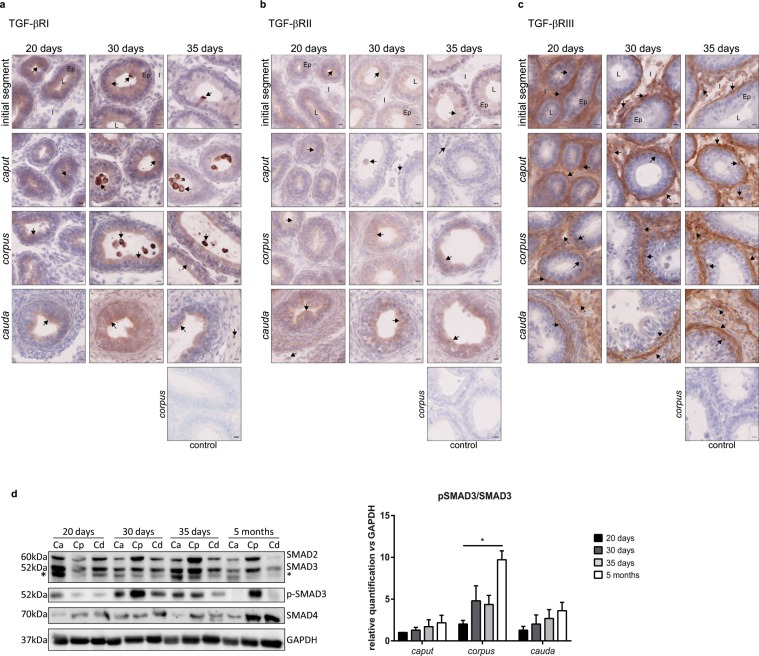
Localisation of the TGF-β receptors and activation status of the signalling pathway in young mice. Localisation of the TGF-β receptors in the initial segment, *caput*, *corpus*, and *cauda* epididymides of young mice, aged from 20 days to 35 days. (**a**) Immunohistochemistry showing TGF-βRI-positive cells. (**b**) Immunohistochemistry showing TGF-βRII-positive cells. (**c**) Immunohistochemistry showing TGF-βRIII-positive cells. The control pictures refer to the *corpus* epididymis from mice aged 35 days. Arrows point to representative stained cells. L: lumen, Ep: epithelium, I: interstitium. Scale bars represent 10 µm. 20 days and 30 days, n = 5; 35 days, n = 4. (**d**) Western blot analysis of the TGF-β signalling pathway proteins in the *caput*, *corpus*, and *cauda* epididymides of young mice (left panel). To allow comparisons, samples from 5 month-old mice were run on the same blots. Blots cropped from different gels are shown. Uncropped versions of the blots are available in Supplementary Fig. S3b. *Shows an aspecific band distinct from the 52 kDa band corresponding to SMAD3. Relative quantification of the ratio between SMAD3 and its phosphorylated form (pSMAD3) normalised with GAPDH (right panel). Bars represent means ± SEM. n = 3, Kruskal-Wallis test, ^*^P < 0.05.

As for the ligands, all three receptors were present throughout the organ by day 20. Until day 30, epithelial staining for TGF-βRI was present in all regions but by day 35, *caput* staining disappeared with the exception of the initial segment. Moreover, a strong staining on stereocils was observed in the *caput* epididymis in young mice, whatever their age. Positive interstitial cells were noted as early as day 20 and were immune cells (Fig. 6a and Supplementary Fig. S4b). Overall, the localisation of TGF-βRI in young mice was similar to that observed in adult mice (Fig. 2b). Epithelial cells were also positive for TGF-βRII but the staining was weak and diffuse in the cytoplasm whatever the age of mice and the region. A few positive interstitial cells were observed on day 20, but only in the *cauda* epididymis. Such cells were then observed in all epididymal regions on days 30 and 35. All TGF-βRII-positive interstitial cells were immune cells (Fig. 6b and Supplementary Fig. S4b). TGF-βRIII-positive epithelial cells, mainly basal cells, were only observed in the *corpus* epididymis of young mice from day 20 to day 35, and some in the *cauda* epididymis on day 20 (Fig. 6c). This result greatly differs from that seen in adult mice. Indeed, 5 month-old mice showed strong epithelial staining (principal and clear cells) throughout the organ (Fig. 2b and Supplementary Fig. S2). On the contrary, interstitial and peritubular TGF-βRIII stainings were similar in young and adult mice (Figs. 2b,c and 6c). Finally, whatever the receptor, positive luminal cells only appeared from day 30.

### The TGF-β signalling pathway in young mice

The activation status of the TGF-β signalling pathway was assessed in young mice by Western blot, as it was in adult mice (Fig. 6d, left panel). No phosphorylation of SMAD2 was observed whatever the region considered (not shown). The pSMAD3/SMAD3 ratio showed a clear increase from day 20 to 5 months even if it failed to reach statistical significance in the *caput* and *cauda* epididymis. However, there was a significant increase in the activation status of the signalling pathway between day 20 and 5 months in the *corpus* epididymis (Fig. 6d, right panel).

## Discussion

In the epididymis, a key determinant of male fertility is the ability to prevent autoimmune responses against immunogenic spermatozoa while providing effective protection against ascending pathogens. Indeed, immune disorders represent up to 15% of known cases of male infertility and this number may be underestimated, as they most likely involve some of the 30% of idiopathic cases^21,22^. Although some immunomodulatory molecules, such as activins^23,24^ and IL-10^25^, have been identified in the epididymis, their roles in the tolerogenic status of the organ have never been proven. Until recently, the immunosuppressive enzyme indoleamine 2,3-dioxygenase 1 (IDO1), which is constitutively expressed by murine epididymal epithelial cells (EECs), was the only molecule that proved to participate in the establishment of an immunological equilibrium within the *caput* epididymis of mice^26,27^. A work published in 2018 provided the first demonstration of a mechanism set in the epididymis to maintain an efficient tolerance to spermatozoa through the TGF-β signalling in dendritic cells^19^. Nevertheless, no data were available concerning the TGF-β isoform(s) implicated in this peripheral tolerance. The present study provides new data regarding the patterns of expression and localisation of the three TGF-β isoforms and of their receptors in the murine epididymis, from pre-pubertal to adult animals.

A striking result was that the active concentration of the three TGF-β isoforms was significantly higher in the *corpus* than in the *caput* and *cauda* epididymis in young and adult mice. Further experiments will be needed to assess the biological significance of this finding but this may reflect a peculiar implication of the *corpus* epididymis in terms of tolerance to sperm cells. This result was correlated with the activation status of the signalling pathway which was particularly high in the *corpus* epididymis compared to the *caput* and *cauda* regions, whatever the age of mice. As activins and TGF-βs both signal through SMAD2/3, one cannot rule out a contribution of activin signalling to the signal detected by Western blot analyses. However, this contribution is not expected to question the importance of the *corpus* region as ACTIVIN A was shown to be homogeneously present throughout the murine epididymis while ACTIVIN B was more present in the *caput* than in the *cauda* region^23,28^. On the contrary, TGF-β signalling may be underestimated in this study because TGF-β signalling pathways independent of SMAD, involving PI3K and various MAP kinases, have been described^29^.

A specific localisation of each TGF-β ligand and receptor was observed along the epididymis, suggesting non-redundant roles for these molecules in the organ. However, whatever the ligand or receptor, three types of staining including epithelial, interstitial, and luminal, were observed by immunohistochemistry. Unfortunately, as the antibodies are produced in the same species, it prevented us from determining the direct interactions existing between each TGF-β isoform and their receptors in the organ.

The epithelial staining is of special interest as epithelium is the first to encounter potential pathogens and spermatozoa. Thus EECs must play a crucial role in both rapid recognition and elimination of pathogens and immunological tolerance to sperm cells. Yet, the ability of EECs to ensure these functions remains largely uncharacterised. The fact that EECs expressed the three TGF-β isoforms in all regions of the murine epididymis in pre-pubertal to adult mice reinforces the idea that the epididymal epithelium plays an active role in establishing a pro-tolerogenic environment necessary for the survival of immunogenic spermatozoa. The presence of p-SMAD3 in nuclei of epithelial cells suggests an active autocrine and/or paracrine signalling in the epididymis. This epithelial signalling could be responsible for the modulation of the blood- epididymal barrier (BEB) permeability as illustrated by an *in vitro* model^30^. Indeed, among the TGF-β isoforms, TGF-β3 was shown to strongly increase the BEB permeability whereas TGF-β1 had a lower impact, and TGF-β2 virtually none. Modulation of the BEB permeability could allow the migration of dendritic cells into the epididymal epithelium, and then the establishment of peripheral tolerance to sperm cells thanks to dendritic cells sampling of luminal sperm antigens. It is interesting to note that the analysis of the “active form/total form” ratios showed that the proportion of active TGF-β3 increased with age whatever the region. As a consequence, almost all TGF-β3 present in adult mice epididymis was of the active form. Moreover, TGF-β3 staining was mainly identified in epithelial cells from early ages to adulthood. This strongly supports the crucial role of this isoform. On the other hand, TGF-β1 has been shown to inhibit both human and mouse dendritic cells maturation *in vitro*, *via* the downregulation of the expression of co-stimulatory molecules^31–33^. It has also been demonstrated that TGF-β1 could decrease the expression of MHC class II molecules on dendritic cells, thus influencing their antigen presentation activity^32^. Thus, it may be interesting to study the maturation state of the epididymal dendritic cells to better understand TGF-β-mediated tolerance. Furthermore, our study showed that TGF-β1 was expressed mainly in its active form in the *caput* and *corpus* epididymis in young and adult mice, suggesting potential cooperation between epididymal TGF-β isoforms. Indeed, TGF-β3 produced by the EECs could be an autocrine/paracrine player on the epithelium to allow the passage of dendritic cells while TGF-β1 produced by the EECs could act on the epididymal dendritic cells to create/maintain a tolerogenic environment. We showed that the active/total form ratio for TGF-β2 was lower than for the other isoforms, however almost half of the TGF-β2 produced in the *corpus* epididymis was in an active form on day 20 and in adult mice, which suggests a potential early role during the epididymal development in young mice in addition to a role in sperm tolerance in adult mice.

In addition to the epithelial production, TGF-β-positive cells were observed in the interstitial and luminal compartments. Although no interstitial TGF-β-positive cells were present in young mice (with the exception of TGF-β1-positive cells which were generally not immune cells), many of them were observed in adult mice, and they were all immune cells. An abundant literature shows a significant production of TGF-β by immune cells to induce tolerance, which supports an important role for these interstitial epididymal cells in maintaining sperm tolerance in adult mice. The CD4^+^ CD25^+^ Foxp3^+^ regulatory T (Treg) cells are the canonical tolerance-inducing cells in peripheral tissues. In mouse, they can be converted from CD4^+^ CD25^−^ Foxp3^−^ T cells by stimulation *via* their T cell receptor in the presence of TGF-β^34,35^, and one of their mechanisms of action is the production of immunosuppressive cytokines, such as IL-10 or TGF-β^36^. Surprisingly, there are no or few Treg cells in the steady state epididymis^19,37^. This discrepancy may be explained by the presence of Foxp3 negative TGF-β-producing regulatory T cell populations, such as Th3, Tr1, or CD4^+^ CD25^−^ LAG3^+^ Treg cells, which have not been investigated yet in the epididymis^38,39^. In addition to regulatory lymphocytes, TGF-β-producing immune cells can be alternatively activated macrophages, *i.e*. of the M2 anti-inflammatory phenotype^40^. This hypothesis is supported by preliminary observations that some interstitial cells expressing the macrophagic marker F4/80 are positive for TGF-β2 in the epididymis of adult mice. Thus, further characterisation is needed to determine the population(s) of TGF-β-producing immune cells in the murine epididymis as it will give clues to their functions in the tolerogenic state of the organ. Finally, TGF-β signalling in the epididymal dendritic cells is known to be crucial to maintain the immune tolerance to spermatozoa in mice but the exact mechanisms remain to be described^19^. It is interesting to note that all interstitial cells positive for TGF-β receptors (with the exception of TGF-βRIII) were immune cells as early as 20 days after birth. In parallel, we found that the concentrations of all three ligands were higher on day 20 than at 5 months, however the signalling pathway in young mice was not as activated as in adult mice. This could be explained by the fact that there are fewer receptors in the epididymis of young mice, which is still to be determined, or by the fact that the level of activation was underestimated in young mice because the signalling is mediated by SMAD-independent pathways, as evoked earlier. These results suggest that conditions are put in place early to allow effective tolerance in the murine epididymis.

Contrary to interstitial cells, TGF-β-positive luminal cells were not immune cells. Their nature needs to be clarified but preliminary data point to some cells positive for the V-ATPase proton pump, suggesting that they are clear cells, at least in part. The V-ATPase-negative cells could either be detached epididymal epithelial cells, different from clear cells, or cells arising from the testis.

TGF-β signalling in the epididymal epithelium may also be involved in mechanisms independent of tolerance, as it could be one of the actors responsible for the low occurrence of primary epididymal cancers. Indeed, epididymal cancers are rare, accounting for only 0.03% of cancers in western countries, whereas the most common male cancer, prostate cancer, represents 20% of cases^41^. Moreover, epididymal cancers are mainly (approximately 80%) benign cancers of epithelial origin. Mechanisms explaining the low rate of tumours in this organ remain largely unknown. It has been suggested that strong antioxidant mechanisms, active tumour suppressors, and inactive oncogenes could prevent the initiation of epididymal tumour development^41^. In addition to these mechanisms, we can hypothesise a contribution of TGF-β signalling in the inhibition of epididymal tumour development, as its tumour-suppressing role is well illustrated by the presence of inactivating mutations in genes encoding TGF-β receptors and SMADs in various human cancers^42–49^. In mouse models, the genetic deletion or downregulation of TGF-β signalling leads to the development of a malignant phenotype in breast, intestine, pancreatic and colon cancers^42^.

In conclusion, this work highlights specific expression and localisation patterns for each TGF-β isoform and receptor, suggesting non-redundant roles in the murine epididymis. Further investigations will be needed to understand the specific role(s) of TGF-β1, TGF-β2 and TGF-β3 in the organ because there may be a close collaboration between them to establish/maintain effective sperm tolerance, as well as complementary mechanisms between epithelial cells and immune cells in the organ. To do so, the Cre-LoxP system could be exploited to generate epididymis-specific knockout mice for each TGF-β isoform^50–52^ even if this technique remains limited since the only Cre recombinase mouse strains available are specific of the *caput* epithelium^53,54^. Given the great implication of the TGF-β signalling in maintaining the tolerance to sperm cells in the murine epididymis, identifying the individual roles of each TGF-β isoform certainly is the first step to understand and potentially treat tolerance breakdowns in the epididymal compartment.

## Methods

### Ethics statement

This study was conducted in accordance with French animal protection law. Animal housing was approved by the local ethics committee (Comité d’Ethique pour l’Expérimentation Animale Auvergne, C2E2A) and validated by the French ministry of higher education, research, and innovation (MESRI) under the accreditation number F-63 014 19.

### Animals

WT BALB/cByJ male mice (Charles River Laboratories, L’Arbresle, France) were housed in an animal facility with controlled environment and fed *ad libitum*. Male adult mice had an average age of 5 months at the time of experiments. Young animals were aged 20 days, 30 days or 35 days. Animals were killed by cervical dislocation and epididymides were collected.

### Elisa

To determine the concentration of each TGF-β isoform in the different compartments of the mouse epididymis, ELISA tests were performed. The fresh epididymides were separated into *caput*, *corpus* and *cauda* regions and weighted before being snap frozen in liquid nitrogen until they were used. Epididymal tissues were then homogenised in tissue extraction buffer (100 mM Tris pH 7.4, 150 mM NaCl, 1 mM EGTA, 1 mM EDTA, 1% Triton X-100, 0.5% Sodium deoxycholate) supplemented with protease (cOmplete™, Roche) and phosphatase inhibitors (1 mM NaF, 1 mM Na_3_VO_4_). Lysates were then maintained under constant agitation for 2 h at 4 °C and centrifuged for 20 min at 13,000 × g at 4 °C. Supernatants were collected and stored at −80 °C until use. The total and active forms of TGF-β were tested using commercial DuoSet ELISA kits purchased from RnDSystems (TGF-β1: DY1679-05; TGF-β2: DY 7346-05; TGF-β3: DY243). Total TGF-β concentration measurement was achieved after activation of the protein extracts according to the manufacturer’s instructions while active TGF-β concentration measurement was achieved without activation. Concentrations were finally expressed as pg/mg tissue. The three isoforms were tested on the same lot of animals.

### Immunostainings

#### Tissue preparation

After dissection, whole epididymides were fixed for 24 h in 4% paraformaldehyde (PFA) at 4 °C, before being processed for embedding either in paraffin or in cryosection medium (Richard-Allan Scientific™ Neg-50™, Microm Microtech).

#### Immunohistochemistry

To localise TGF-β isoforms and receptors, and to localise cells with an active TGF-β signalling pathway, immunohistochemistry was performed. Cryosections (10 µm, TGF-β isoforms and receptors) were fixed for 15 min in 4% PFA and paraffin sections (5 µm, phospho-SMAD3) were deparaffinized and rehydrated before antigen retrieval as described in Supplementary Table S1. After endogenous peroxidases inhibition with 0.3% H_2_O_2_, slides were blocked for 1 h, and incubated with primary antibodies, overnight (O/N) at 4 °C. Slides were further incubated with the ImmPRESS™ Horseradish Peroxidase reagent kit (MP7401, Vector Laboratories) or with a biotin-goat anti-rabbit IgG antibody (1:500, 111-065-003, Jackson Immunoresearch) followed by an incubation with peroxidase-conjugated streptavidin (1:500, 016-030-084, Jackson Immunoresearch) for the TGF-β1 staining only. Color was developed with the VECTOR NovaRED peroxidase substrate kit (Vector Laboratories). Slides were then counterstained with Mayer’s hematoxylin for 10 s, mounted in PBS-Glycerol (v/v) before observation with a Carl Zeiss Axio Scan.Z1. Digital images were processed with the Zen software (Carl Zeiss).

#### Immunofluorescence

To identify the cells positive for TGF-β and TGF-β receptors, costaining was performed using a pan-leucocyte marker (CD45). Cryosections (10 µm) were fixed for 15 min in 4% PFA and endogenous peroxidases were inhibited with 0.3% H_2_O_2_. They were then processed as described in Supplementary Table S1. Briefly, after a 1 h blocking step, slides were incubated with primary antibodies, overnight (O/N) at 4 °C. Slides were further incubated with the ImmPRESS™ Horseradish Peroxidase reagent kit (MP7401, Vector Laboratories), and an A555-labelled tyramide (B40955, ThermoFisher Scientific) according to the manufacturer’s instructions or an A555 goat anti-rabbit antibody (2 μg/ml, A21430, ThermoFisher Scientific) for the TGF-β1 staining only. For the co-localisation with CD45, sections were further saturated for 1 h and incubated with the anti-CD45 primary antibody (550539, BD Biosciences) O/N at 4 °C before incubation with an A488-goat anti-rat IgG (2 μg/ml, A11006, ThermoFisher Scientific). Finally, slides were counterstained with 1 μg/ml Hoechst 33342, mounted in Citifluor™ Tris-MWL 4-88 solution and observed with a Leica SPE confocal microscope. Digital images were processed with the OMERO open-source software. All the antibodies shown were tested on the same lot of animals.

### Quantification of mRNA by Real-Time quantitative RT-PCR

Total mRNAs from snap frozen *caput*, *corpus* or *cauda* epididymides were isolated using the NucleoSpin RNA II column kit (Macherey-Nagel), according to the manufacturer’s instructions. RNA (0.5 μg) was reverse transcribed with Moloney murine leukemia virus (MMLV) reverse transcriptase and random hexamer primers (Promega) according to the manufacturer’s instructions. Quantitative PCR was performed with SYBR Premix Ex Taq II (TaKaRa Bio) on a LightCycler 480 II (Roche). Primer sequences are listed in Supplementary Table S2. The relative accumulation level of each mRNA was normalised using *36b4* as a standard, using the comparative Ct method (ΔΔCt method). All PCR were done on the same lot of animals.

### Western blot analyses

To assess the activation status of the TGF-β signalling pathway in the murine epididymis, Western blot analyses were performed. Upon recovery, epididymides were separated into *caput*, *corpus* and *cauda* regions and snap frozen until use. They were then homogenised in high salt buffer (25 mM HEPES, 0.4 M NaCl, 1.5 mM MgCl_2_, 0.2 mM EDTA, 1% IGEPAL^®^ CA-630) supplemented with a protease inhibitor cocktail (cOmplete™, Roche) and phosphatase inhibitors (1 mM NaF, 1 mM Na_3_VO_4_). Lysates were kept on ice for 20 min and centrifuged at 4 °C for 10 min at 13,000 × g. Supernatants were collected and the proteins were dosed by the Bradford method^55^. For electrophoresis, 40 μg (Fig. 3) or 15 µg (Fig. 6) of proteins were diluted in Laemmli sample buffer and incubated for 5 min at 95 °C. Proteins were separated by SDS-PAGE and transferred onto nitrocellulose membranes (Hybond ECL, GE Healthcare). Blots were blocked with Tris Buffered Saline (TBS) (50 mM Tris, 150 mM NaCl) containing 5% low-fat dried milk/0.1% Tween 20 (for the anti-glyceraldehyde 3-phosphate dehydrogenase [GAPDH] antibody) or 5% bovine serum albumin (BSA)/0.1% Tween 20. Membranes were probed overnight at 4 °C with either anti-GAPDH (1:20,000; G9545 Sigma-Aldrich), anti-SMAD2 (1:500, Fig. 3), anti-SMAD3 (1:500, Fig. 3), anti-SMAD2/3 (1:500, Fig. 6), anti-SMAD4 (1:500), anti-phospho-SMAD2 (1:500) or anti-phospho-SMAD3 (1:500) (SMAD2/3 Antibody Kit Sampler 12747, Cell Signaling Technology) antibodies in corresponding blocking solutions. After washing, membranes were incubated with a goat anti-rabbit horseradish peroxidase-conjugated secondary antibody (1:10,000 [GAPDH] or 1:1,000 [others], BI 2407, Abliance) for 1 h at room temperature (RT). Detection was performed using the Clarity^TM^ Western ECL Substrate (Biorad) on a ChemiDoc^TM^ MP Imaging System (Biorad). To see the full-length blots, please refer to the Supplementary Fig. S3. Densitometric analyses were carried out using Image lab software (Biorad). Intensity of the single band corresponding to SMAD3 or to its phosphorylated form p-SMAD3 was normalised with the intensity of the GAPDH band, before the p-SMAD3/SMAD3 ratio was calculated. All antibodies were tested on the same animals.

### Statistical analysis

A non-parametric Kruskal-Wallis test with Dunn’s post-test was used to determine whether the experimental differences were statistically significant. In experiments on adult mice (Figs. 1–3), differences between the epididymal regions were analysed while in experiments showing young animals (Figs. 4–6), differences between mice ages were analysed. A *P* value of 0.05 or less was considered statistically significant. The software GraphPad PRISM v6.01 was used to perform the statistical analyses and to generate the associated graphs.

## Supplementary information

Supplementary dataset.
