# Supplementary material for: Differential expression and localisation of TGF-β isoforms and receptors in the murine epididymis
Source: Sci Rep. 2020 Jan 22;10:995. doi: 10.1038/s41598-020-57839-5 (PMC6976608; doi:10.1038/s41598-020-57839-5)
Supplement: Supplementary file 1 — Supplementary dataset. [file 41598_2020_57839_MOESM1_ESM.pdf]

Differential expression and localisation of TGF-β isoforms and receptors in the murine epididymis.

Allison Voisin, Christelle Damon-Soubeyrand, Stéphanie Bravard, Fabrice Saez, Joël R. Drevet, Rachel Guiton

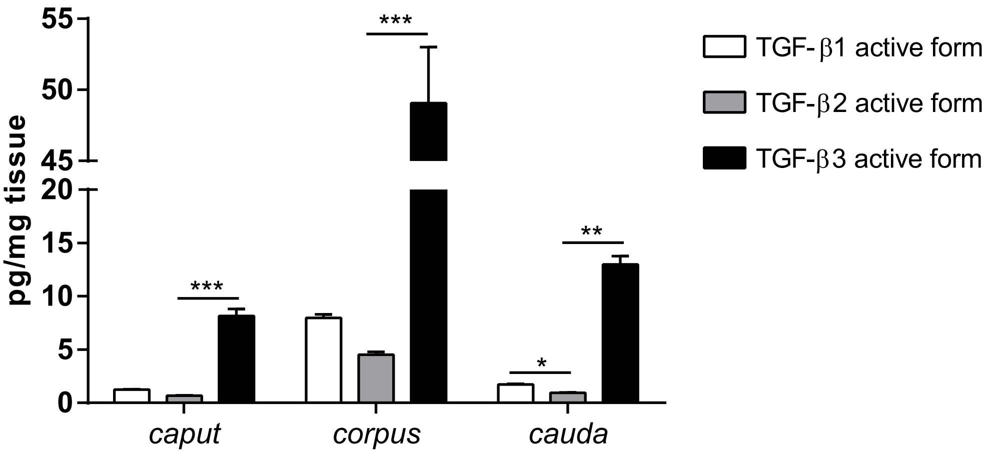

Supplementary fig. S1: Quantification of the active forms of TGF-β isoforms in adult mice.

Concentrations of the active form of the three TGF-β isoforms were determined by ELISA (pg/mg tissue) in the caput, corpus, and cauda epididymides of 5 month-old mice. Bars represent means ± SEM. n=7 (TGF-β1 and β2), n=5 (β3). Kruskal-Wallis test, *$P < 0.05$, **$P < 0.01$, ***$P < 0.001$.

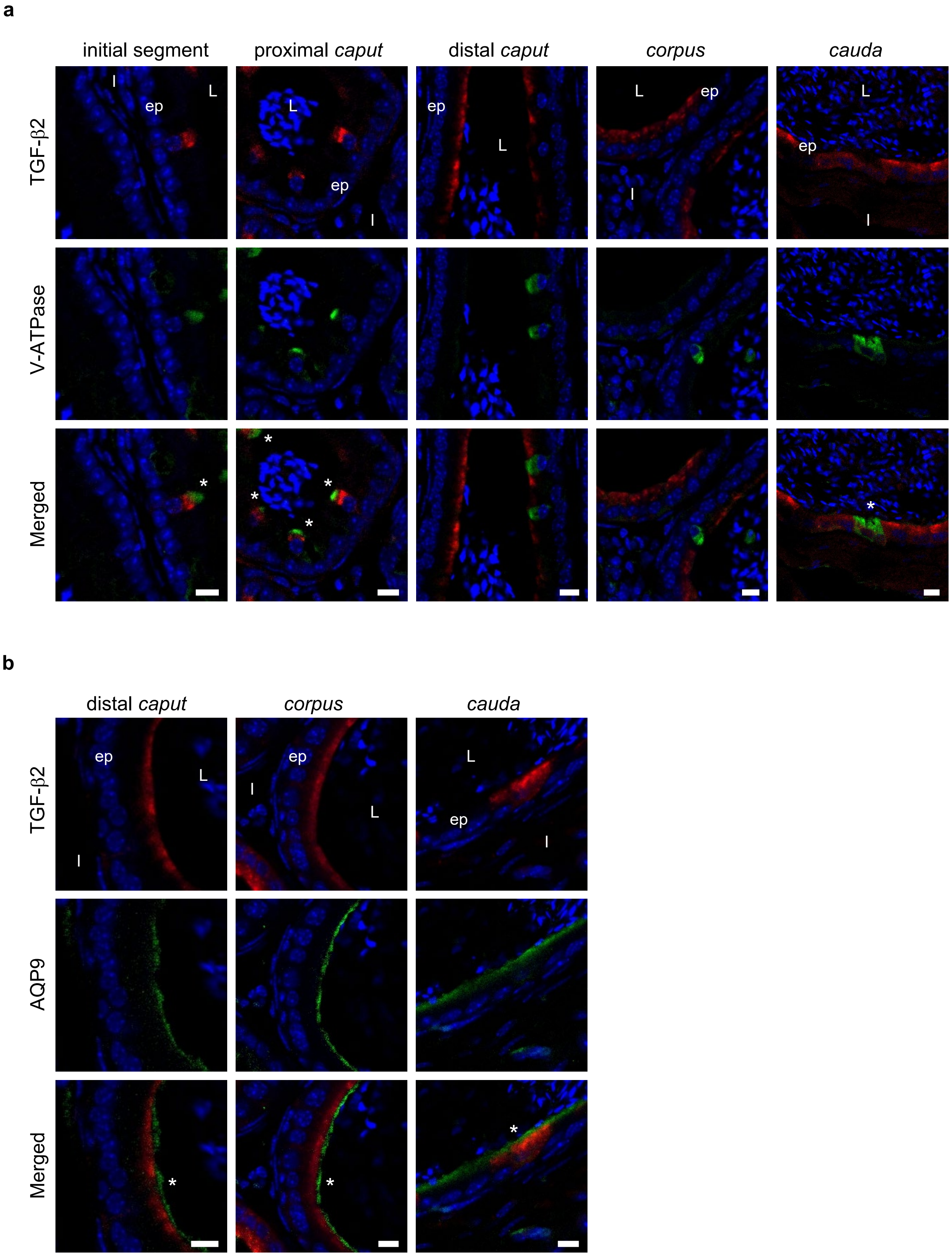

Supplementary fig. S2: Identification of the TGF-β-producing cells in the epididymal epithelium of adult mice.

Immunofluorescence staining showing TGF-β2 (red) and V-ATPase or AQP9 (green) to identify clear cells and principal cells, respectively, in the epididymis of 5 month-old mice. * show co-stained cells. Co-staining was performed for all TGF-β isoforms and receptors.
L: lumen, ep: epithelium, I: interstitium, Scale bars represent 10µm. n=5.

a

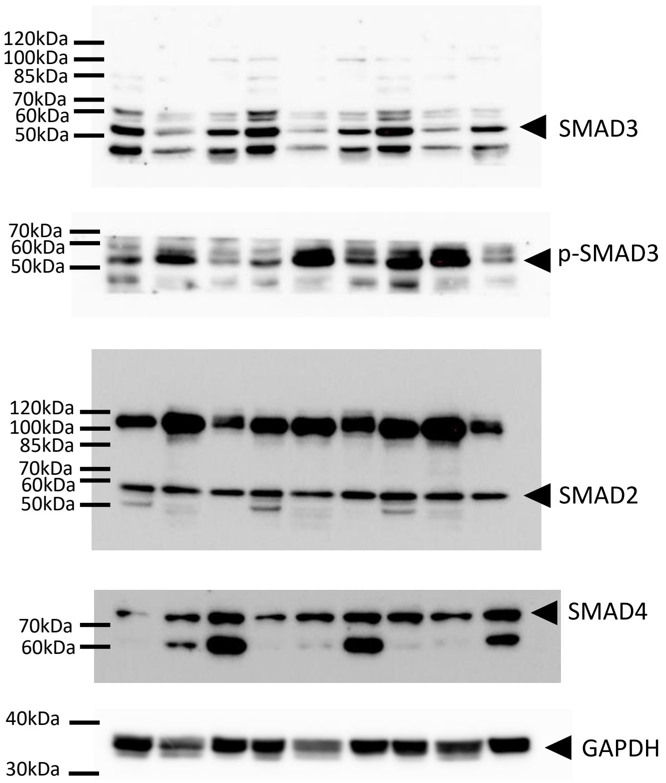

b

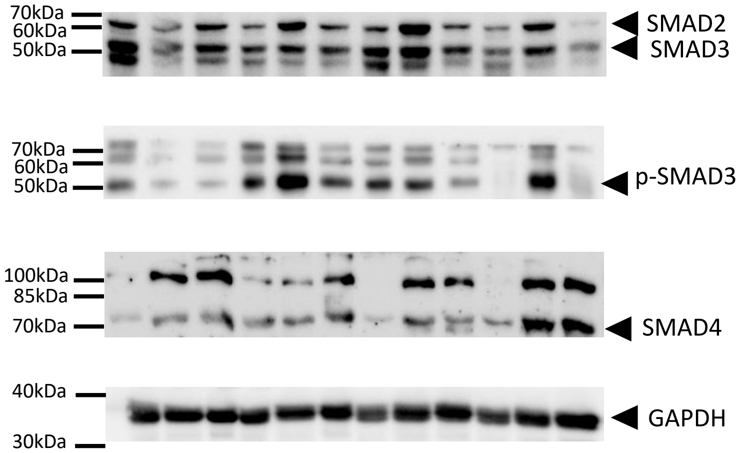

Supplementary fig. S3: Uncropped versions of the western blots shown in Fig.3b (a) and Fig. 6d (b)

.

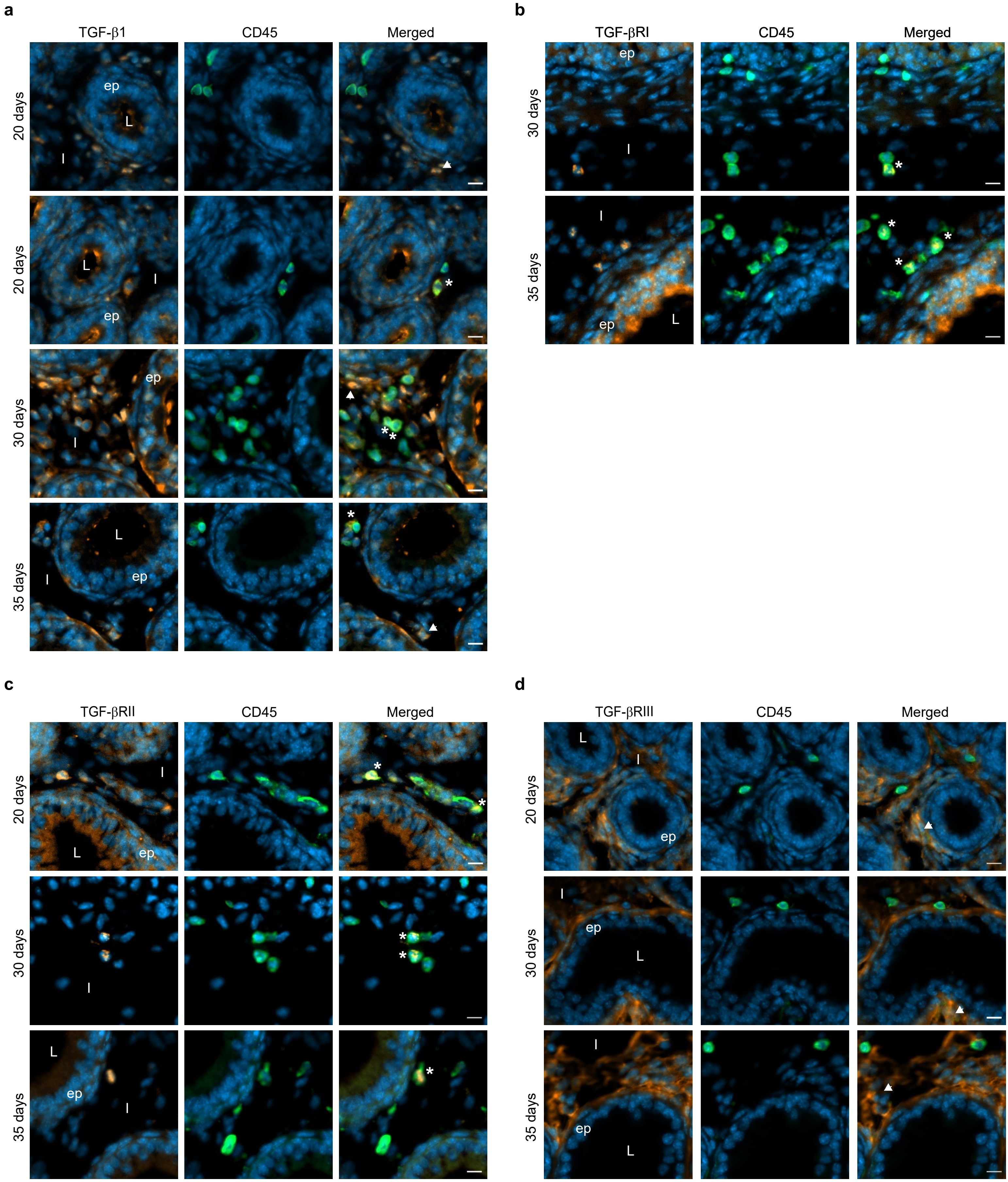

Supplementary fig. S4: Identification of TGF-β isoforms and receptors positive cells in the epididymal interstitium of young mice.

TGF-β1 (a), TGF-βRI (b), TGF-βRII (c) and TGF-βRIII (d) interstitial positive cells (A555, red) were identified based on their expression of the surface marker CD45 (pan-leucocyte, green) in 20, 30 and 35 day-old mice. *show co-stained cells, ▶ show cells negative for the cd45 marker.

L: lumen, ep: epithelium, I: interstitium, Scale bars represent 10μm. n=5 (20 and 30 days), n=4 (35 days).

Antibody	Supplier	Reference	Host	Epitope retrieval	Permeabilisation	Blocking	Application
TGF-β1	Abcam	Ab92486	Rabbit	None	Triton 0.1 %	2.5 % HS (IHC); 5 % HS/1 % BSA (IF)	IHC (1:300), IF(1:100) cryosections
TGF-β2	Santa Cruz	sc-90	Rabbit	None	Triton 0.1 %	2.5 % HS	IHC and IF (1:200) cryosections
TGF-β3	Novus Biological	NB600-1531	Rabbit	None	Triton 0.1 %	2.5 % HS	IHC and IF (1:200) cryosections
TGF-βRI	Abcam	Ab31013	Rabbit	None	Triton 0.1 %	2.5 % HS	IHC and IF (1:100) cryosections
TGF-βRII	Abcam	Ab61213	Rabbit	None	Triton 0.1 %	5 % HS/ 1 % BSA	IHC (1:50), IF(1:200) cryosections
TGF-βRIII	RnD Systems	AF5034	Goat	None	Triton 0.1 %	2.5 % HS	IHC and IF (1:200) cryosections
CD45	BD Biosciences	550539	Rat	None	None	2.5 % HS	IF (1:100) cryosections
phospho-SMAD3	Abcam	Ab52903	Rabbit	25min sodium citrate 10mM, Tween 0.05%	None	2.5 % HS/ 1 % BSA	IHC (1:100) paraffin sections

Supplementary table S1: Antibodies and conditions used for immunostainings.
BSA: Bovine Serum Albumin, HS: Horse Serum, IF: immunofluorescence, IHC: Immunohistochemistry.

Gene	Primer sequences (5'-3')	
	Forward	Reverse
Tgf-βRI	TGCTGCAATCAGGACCACTG	TGGTGAATGACAGTGCCGGTT
Tgf-βRII	GAGTCGTTCAAGCAGACGGA	GAACCAAATGGGGGCTCGTA
Tgf-βRIII	CCCTGCATCTGAACCCCATT	GACCACAGAACCCTCCGAAA

Supplementary table S2: Primer sequences used to determine the expression level of the three TGF-β receptors by qRT-PCR.

Supplementary methods

Immunofluorescence shown in supplementary figure S2

The immunofluorescent stainings were performed on cryosections (10 µm). TGF-β2 staining was performed as described in the materials and methods section. Then slides were treated with 0.02 N HCl for 20 min, washed and epitope retrieval performed in PBS containing 1 % SDS (v/v) for 4 min. After 1h of blocking in 2.5 % horse serum, slides were incubated with the anti-V-ATPase antibody (1:100, gift from Dr. S. Breton, Boston, USA) or the anti-AQP9 antibody (1:100, Merck AB3091) O/N at 4 °C. The V-ATPase-stained sections were further incubated with an A488 anti-chicken IgG. The AQP9-stained sections were incubated with a biotin anti-chicken IgG (1:500, Bethyl laboratories) followed by an incubation with peroxidase-conjugated streptavidin (1:500, Jackson Immunoresearch) and an A555-labelled tyramide (ThermoFisher Scientific), according to the manufacturer's instructions. Finally, slides were counterstained with 1 µg/ml Hoechst 33342, mounted in Citifluor™ Tris-MWL 4-88 solution and observed with a Leica SPE confocal microscope. Digital images were processed with the OMERO open-source software.
